# Impact of integrated district level mental health care on clinical and functioning outcomes of people with depression and alcohol use disorder in Nepal: a non-randomised controlled study

**DOI:** 10.1186/s12888-020-02832-5

**Published:** 2020-09-14

**Authors:** M. J. D. Jordans, E. C. Garman, N. P. Luitel, B. A. Kohrt, C. Lund, V. Patel, M. Tomlinson

**Affiliations:** 1 Centre for Global Mental Health, Health Service and Population Research Department, Institute of Psychiatry, Psychology and Neuroscience, King’s College London, London, UK; 2 Transcultural Psychosocial Organization (TPO), Nepal, Kathmandu, Nepal; 3 Alan J Flisher Centre for Public Mental Health, Department of Psychiatry and Mental Health, University of Cape Town, Cape Town, South Africa; 4 Department of Psychiatry, George Washington University, Washington, DC, USA; 5 Harvard Medical School, Boston, USA; 6 Sangath, Porvorim, Goa India; 7 Harvard Chan School of Public Health, Boston, USA; 8 Institute for Life Course Health Research, Department of Global Health, Stellenbosch University, Stellenbosch, South Africa; 9 School of Nursing and Midwifery, Queens University, Belfast, UK

**Keywords:** Primary health care, Community mental health, Nepal, Low and middle income countries, Psychological treatment

## Abstract

**Background:**

Integration of mental health services into primary healthcare is proliferating in low-resource countries. We aimed to evaluate the impact of different compositions of primary care mental health services for depression and alcohol use disorder (AUD), when compared to usual primary care services.

**Methods:**

We conducted a non-randomized controlled study in rural Nepal. We compared treatment outcomes among patients screening positive and receiving: (a) primary care mental health services without a psychological treatment component (TG); (b) the same services including a psychological treatment (TG + P); and (c) primary care treatment as usual (TAU). Primary outcomes included change in depression and AUD symptoms, as well as disability. Disability was measured using the 12-item WHO Disability Assessment Schedule. Symptom severity was assessed using the 9-item Patient Health Questionnaire for depression, the 10-item Alcohol Use Disorders Identification Test for AUD. We used negative binomial regression models for the analysis.

**Results:**

For depression, when combining both treatment groups (TG, *n* = 77 and TG + P, *n* = 60) compared to TAU (*n* = 72), there were no significant improvements. When only comparing the psychological treatment group (TG + P) with TAU, there were significant improvements for symptoms and disability (aβ = − 2.64; 95%CI − 4.55 to − 0.74, *p* = 0.007; aβ = − 12.20; 95%CI − 19.79 to − 4.62; *p* = 0.002, respectively). For AUD, when combining both treatment groups (TG, *n* = 92 and TG + P, *n* = 80) compared to TAU (*n* = 57), there were significant improvements in AUD symptoms and disability (aβ = − 15.13; 95%CI − 18.63 to − 11.63, *p* < 0.001; aβ = − 9.26; 95%CI − 16.41 to − 2.12, *p* = 0.011; respectively). For AUD, there were no differences between TG and TG + P. Patients’ perceptions of health workers’ skills in common psychological factors were associated with improvement in depression patient outcomes (β = − 0.36; 95%CI − 0.55 to − 0.18; *p* < 0.001) but not for AUD patients.

**Conclusion:**

Primary care mental health services for depression may only be effective when psychological treatments are included. Health workers’ competencies as perceived by patients may be an important indicator for treatment effect. AUD treatment in primary care appears to be beneficial even without additional psychological services.

## Background

Only 1 in 27 people with depression receive minimally adequate care in low- and middle-income countries (LMIC) [2]. This treatment gap, defined as the difference between people in need of mental health services and those actually accessing such services, is similarly pronounced for any substance use disorder [3]. In an effort to address this problem, the World Health Organization (WHO) has developed guidance for primary healthcare workers to provide first-line mental health care [4]. Task-sharing mental health care from specialists to primary healthcare workers aims to increase access to care. Previous research has evaluated mental health services delivered by non-professionals, synthesized in a series of recent reviews, demonstrating positive outcomes of this approach [5–7]. In the current study, we aimed to evaluate this approach in Nepal, and add to this body of literature by unpacking what, how and for whom task-shared services are most beneficial. In Nepal, one out of five women attending primary care services screen positive for depression and one out of five men attending primary care services screen positive for Alcohol Use Disorder (AUD) [8].

At present there is little evidence regarding what implementation strategies for the above-mentioned task-shifting approach, using WHO’s mental health Gap Action Programme (mhGAP) Intervention Guidelines (IG), are effective in supporting people with depression and AUD [9]. The Programme for Improving Mental Health Care (PRIME) aimed to address this evidence gap [10]. PRIME developed and evaluated a population-level mental health care plan for people with depression, AUD, epilepsy and psychosis in five LMICs. In Nepal, the package included interventions targeting the community (i.e. awareness raising, counselling services and proactive case detection), healthcare workers (i.e. detection, diagnosis and treatment following mhGAP IG), and the health system (i.e. referral services, supervision) [11, 12].

Our primary analyses of the integration of mental health into primary care demonstrated that patients with depression and AUD showed improvement in symptoms and reduced disability 1 year after initiating treatment (effect size d = 0.34 for AUD; d = 0.58 for depression) [13]. In addition, a randomised controlled trial was embedded in the study, which means that, among those who were enrolled in the treatment cohorts, half were randomised to receive additional psychological treatment by a separate cadre of service providers: the Healthy Activity Programme (HAP) for those in the depression treatment cohort or Counselling for Alcohol Problems (CAP) for those in the AUD treatment cohort [14]. HAP and CAP are manualised individual interventions delivered over several weeks, which make use of behavioural activation and motivational interviewing techniques, respectively. We found that patients with depression randomized to receive primary care worker mental health services plus the manualized psychological intervention showed greater improvement compared to patients only receiving the primary care mental health services without a psychological treatment. In contrast, we found that patients with AUD improved comparably whether they received only primary care mental health services or primary care services plus a manualized psychological treatment [14].

However, our prior analyses did not compare outcomes among these patients compromising a control group with patients who screen positive for depression or AUD who did not receive a diagnosis and treatment from primary care workers. Therefore, the goal of the current analysis was to compare how patients receiving different constellations of treatment compared with patients receiving treatment as usual in primary care without the integrated mental health services. Moreover, we explored how patients’ perceptions of quality of care and health workers’ skills in common factors (e.g., empathy, promoting hope, communication skills) were associated with treatment outcomes. Such analyses will help in better understanding for whom and how task-shifted mental health care by primary health care workers might contribute to patient improvements.

## Methods

### Context

This study was part of a multi-country research program that implemented and evaluated district level-mental health care plans (MHCP) in Ethiopia, India, Nepal, South Africa and Uganda (Lund et al., 2012). In Nepal, the program was implemented in Chitwan, a district with a population of nearly 580,000 in the south of the country. Before PRIME, no mental health services were available in primary care settings, and were instead restricted to specialized settings.

### Design

To assess the impact of the MHCP on the clinical and functional outcomes of individuals with depression and AUD, patients were recruited into cohorts and followed up twice until 12 months post-recruitment. A comprehensive description of study methods has already been published [15]. A brief overview of the methods employed are described here.

### Sample and recruitment

Individuals eligible for the study were patients attending one of the 10 facilities where the MHCP was implemented, who were 16 years or older, lived in the district and were willing and able to provide informed written consent. Participants were informed about the nature of the study and about their right to withdraw from the study at any point in time. Consenting patients were screened by PRIME research assistants before their consultation with a primary health care worker, using the Patient Health Questionnaire (PHQ-9) [1] and the Alcohol Use Disorder Identification Test (AUDIT) [16]. Participants who scored above the instrument cut-off on either PHQ-9 or AUDIT were interviewed again by the research assistant after their consultation with the primary care worker.

**Fig. 1:**
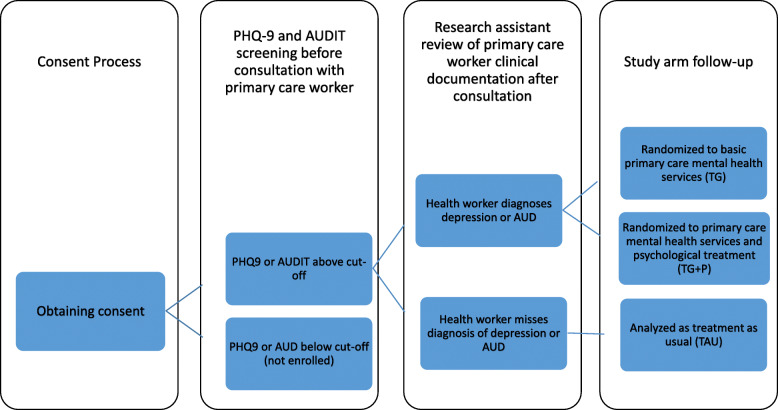
Overview study design

Patients scoring above one of these cut-offs and who were diagnosed by the primary care worker with either depression or anxiety were subsequently randomized to receive mental health services without a psychological treatment component (i.e. ‘treatment group, TG’; *n* = 77 for depression, *n* = 92 for AUD); or to receive primary mental health care services including a manualized psychological treatment, (i.e. ‘treatment with psychological services group, TG+P’; *n* = 60 for depression, *n* = 80 for AUD). Patients scoring above one of the cut-offs and whose diagnosis was missed by the primary care worker were classified as the ‘treatment as usual (TAU; *n*=72 for depression, *n*=57 for AUD)’, serving as the control group (see Fig. 1). The sample has not been included in prior studies. Participants who received a dual diagnosis were enrolled in the AUD cohort. Likewise, a participant was recruited into the AUD control group if they screened positive on both PHQ-9 and AUDIT without primary care worker diagnosis. We were aware that the participants between the treatment arms (TG and TG + P) and the control arm (TAU) differed, in that the former consists of participants diagnosed by health workers and the latter group of participants are not diagnosed. Still, we considered this comparison to be a helpful strategy to identify what treatment components and treatment perceptions are predictors of clinical and functional outcomes. A sample size of 200 participants in the depression and AUD cohort was considered sufficient to detect a 20% reduction in symptom severity at the 12-month follow-up among the treatment groups, with a 90% power and two-sided alpha at 0.05. This sample size calculation also took into account an attrition rate of 15–20%.

### Intervention

The MHCP that has been developed and implemented in Nepal, in partnership with the Ministry of Health, has been described in detail elsewhere [12]. In brief, it comprised interventions at the community, health facility and health service organisation levels. The community level interventions included community sensitization, pro-active case detection by female community health volunteers [17], and home-based care to boost treatment adherence. In addition, community counsellors delivered the Healthy Activity Program (HAP) for depression, consisting of 6–8 weekly sessions that include behavioural activation as the core therapeutic framework [18], and Counselling for Alcohol Problems (CAP) for AUD, a manualized motivational interviewing intervention of 4 sessions delivered weekly [19]. The facility-level interventions included training of health workers to detect, diagnose and initiate treatment (i.e. emotional support, psycho-education and psychotropic medication) for those diagnosed with one of the priority disorders, following the mhGAP Intervention Guide for primary health workers [4]. Finally, health service organisation level interventions included ensuring availability of psychotropic medication and referrals to specialised care. For all clinical services, regular ongoing supervision was part of the MHCP. Participants enrolled in the control group received healthcare as usual, consisting of assessment, diagnosis and treatment of somatic conditions.

### Instruments

All participants were assessed on a range of demographic and socio-economic measures. The primary outcome measure for both cohorts was disability, measured using the 12-item WHO Disability Assessment Schedule (WHODAS 2.0) [20]. The WHODAS 2.0 has been validated in a range of settings [21], and has previously been used in Nepal [22, 23]. The item-response-theory (IRT) scoring method was used, so scores ranged from 0 to 100; the higher the score the greater the impairment. The other primary outcome was symptom severity, assessed with the PHQ-9 in the depression cohort and the AUDIT in the AUD cohort.

The PHQ-9 is a 9-item questionnaire which was validated in Nepal as part of PRIME’s formative phase and has shown to be a reliable and valid tool to assess depressive symptoms in that population [24]. Scores range from 0 to 27, with greater scores indicating more severe depressive symptoms. According to the validation study, a cut-off of 10 was identified as the optimal score to indicate high risk for depression, and was the score used to identify participants eligible to be recruited into the depression control group in the present study.

The AUDIT is a 10-item questionnaire which assesses alcohol misuse on a scale of 0 to 40; again, higher scores indicate more severe symptoms. The AUDIT has been validated in Nepal, where it was found to be a reliable measure to identify dependent and hazardous drinkers [25]. A cut-off of 9 was suggested to identify both women and men at risk for alcohol abuse, which was the cut-off used in the present study to identify individuals eligible for the AUD control group.

The 20-item Patient Assessment on Chronic Illness Care (PACIC) is a self-report tool to assess perceived quality of patient-centred care for chronic illness consistent with the Chronic Care Model [26]. The items are scored on a 5-point likert scale with 1 being “None of the time” and 5 being “Always”. In addition, patients rated the health worker’s empathy, therapeutic alliance, psychoeducation, communication, and other skills. These skills are collectively referred to as common factors in psychological treatment research. A tool has been developed in Nepal to assess common factors: the ENhancing Assessment of Common Therapeutic factors (ENACT) scale is an 18-item instrument for the assessment of common factors of clinical competency [27]. A patient 15-item report version has also been developed in which patients rate their health worker on domains including empathy, providing clear explanations, not embarrassing the patient, promoting hope for recovery, eliciting feedback, and an appropriate mobilization of family or social support.

### Procedure

Participants were assessed three times: at recruitment (baseline), and again after 3 months (midline) and 12 months (endline). The baseline assessment was initiated at the health facility where participants were recruited, and when needed because of limited availability, was completed at the participants’ homes. Thereafter, all assessments were conducted in the participants’ homes. Data collection was completed using android devices linked to an online application (www.mobenzi.com), which allowed for real-life scoring and minimised human error or missing data. Fieldworkers were given a two-week and four-week window before and after midline and endline, respectively, to complete the assessment. Fieldworkers considered participants lost to follow-up after three attempts to schedule an assessment. Participants deemed to be at imminent risk for self-harm or suicide (i.e. a positive response to questions on suicidal ideation and either recent attempt or current plans for suicide) were referred to professional mental health services.

### Analysis

Data were exported from the online data collection application into Stata 14, where the data were analysed. All analyses were conducted separately for the depression and AUD cohorts. Measures of central tendencies were used to summarise the sample characteristics in both cohorts. None of the PHQ-9, WHODAS or AUDIT scores were normally distributed, and so medians and interquartile ranges are reported instead. Since participants were not randomly assigned to the control and treatment groups within each cohort, non-parametric tests were performed to identify differences between the groups on demographic, socio-economic and health-related characteristics (Fisher’s Exact Test for categorical variables and Wilcoxon rank-sum tests for continuous variables). Variables on which the treatment and control groups differed (*p* < 0.05) were then entered in a subsequent model as covariates to generate the probability of being allocated to the treatment group. The inverse of that probability was then used as a weight in all subsequent analyses, to take into account baseline differences in sample characteristics [28].

Negative binomial regression models were used to assess the effect of treatment on participants’ functioning and symptom severity over time, in the depression and AUD cohort separately. This type of model was employed to overcome the highly skewed distributions of the WHODAS, PHQ-9 and AUDIT scores. Post-hoc analyses involved the same approach, this time comparing participants in the control groups with only those in the treatment cohorts who also received either HAP or CAP interventions. Effect sizes (Cohen’s d) for the mean difference in change in outcome scores between the control and treatment groups are presented.

To assess the association of the participants’ perceived quality of care and perceived health worker competency with outcomes, negative binomial regressions were run again among participants in the treatment cohorts only, this time including scores on the patient-ENACT and PACIC as interaction terms with time in two separate models.

## Results

**Fig. 2:**
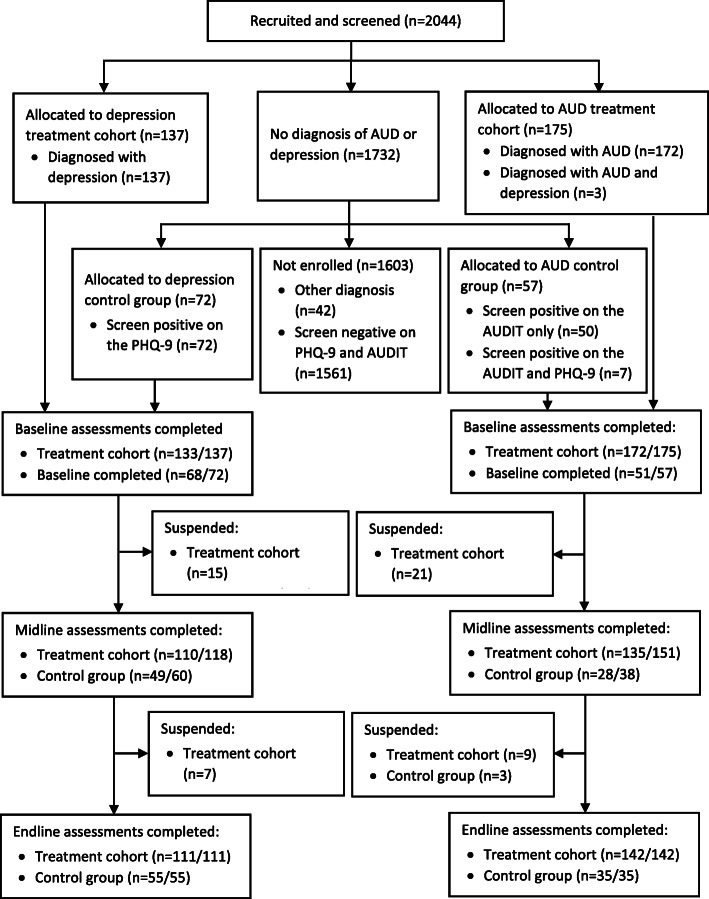
Flow diagram illustrating the recruitment and data collection process

A total of 2044 patients were recruited into the study and screened with the PHQ-9 and AUDIT. Of these, 137 and 172 were diagnosed with depression and AUD, respectively (see Fig. 2). An additional 3 participants received a dual diagnosis and were recruited in the AUD treatment cohort. Of those not receiving a diagnosis of depression, AUD or another PRIME priority condition, 72 screened positive on the PHQ-9, 50 screened positive on the AUDIT, and 7 screened positive on both screening tools. A total of 72 and 57 participants were then recruited in the depression and AUD control groups, respectively.

Table 1 reports the sample characteristics in the depression and AUD cohorts. Participants in the depression cohort were on average 41 years old (SD = 14.1); the majority of participants were female (*n* = 181, 86.6%), unemployed (*n* = 142, 73.2%), Hindu (*n* = 176, 84.2%) and reported having a partner (*n* = 175, 83.7%). Fisher’s Exact tests indicated that the treatment and control cohorts only differed on caste: a greater proportion of participants in the treatment cohort reported being from the Brahmin/Chhetri caste (*n* = 59, 53.1%), whereas a greater proportion of participants in the control group reported being from the Dalit caste (*n* = 32, 44.4%; *p* = 0.007). Participants in the treatment cohort also reported greater WHODAS scores (median = 36, interquartile range (IQR) = 22–47) compared to participants in the control group (median = 29.5, IQR = 20.5–36); a significant difference based on a Wilcoxon rank-sum test (U = -2.49; *p* = 0.013).

**Table 1:** Baseline demographic and clinical characteristics of the cohort samples

	Depression cohort (*N* = 209)						AUD cohort (*N* = 232)					
	All		Control (*n* = 72)		Treatment (*n* = 137)		All		Control (*n* = 57)		Treatment (*n* = 175)	
	n	%	N	%	n	%	n	%	n	%	n	%
Female	181	86.6	63	87.5	118	86.1	31	13.4	5	8.8	26	14.9
Has a partner	175	83.7	64	88.9	111	81.0	215	92.7	50	87.7	165	94.3
16–30 years old	55	26.3	23	31.9	32	23.4	30	12.9	7	12.3	23	13.1
30–50 years old	105	50.2	36	50.0	69	50.4	137	59.1	29	50.9	108	61.7
> 50 years old	49	23.4	13	18.1	36	26.3	65	28.0	21	36.8	44	25.1
Uneducated/illiterate	55	26.3	17	23.6	38	27.7	46	19.8	9	15.8	37	21.1
Less than primary school	58	27.8	20	27.8	38	27.7	50	21.6	17	29.8	33	18.9
Primary school & above	96	45.9	35	48.6	61	44.5	136	58.6	31	54.4	105	60.0
Lowest wealth	53	33.3	17	34.0	36	33.0	62	33.0	10	25.6	52	34.9
Middle wealth	53	33.3	17	34.0	36	33.0	63	33.5	20	51.3	43	28.9
Highest wealth	53	33.3	16	32.0	37	34.0	63	33.5	9	23.1	54	36.2
Employed	52	26.8	15	23.1	37	28.7	176	79.3	42	82.3	134	78.4
Hindu	176	84.2	58	80.6	118	86.1	182	78.5	48	84.2	134	76.6
Brahman/Chhetri	80	38.3	21	29.2	59	53.1	75	32.3	13	22.8	62	33.4
Dalit	64	30.6	32	44.4	32	23.4	74	31.9	24	42.1	50	28.6
Janajati and other	65	31.1	19	26.4	46	33.6	83	35.8	20	35.1	63	36.0
	Med.	IQR	Med.	IQR	Med.	IQR	Med.	IQR	Med.	IQR	Med.	IQR
PHQ-9 score	12	10–15	11	11–13	12	10–15	7	4–11	4	3–7	8	5–12
AUDIT score	0	0–1	0	0–0	0	0–1	24	21–30	15	11–21	27	21–30
WHODAS score	33	22–44	29.5	20.5–36	36	22–47	17	8–28	14	6–25	19	8–31

*AUD* Alcohol use disorder, *AUDIT* Alcohol use disorder identification test, *Med.* Median, *PHQ-9* Patient Health Questionnaire – 9 item, *WHODAS* WHO Disability Assessment Schedule

In the AUD cohort, participants were on average 39 years old (SD = 14.0), and primarily male (*n* = 215, 92.7%) and employed (*n* = 176, 79.3%). Similarly to the depression cohort, the majority of participants in the AUD cohort reported having a partner (*n* = 215, 92.7%) and were Hindu (*n* = 182, 78.5%). Fisher’s Exact test showed that a significantly greater proportion of participants in the control group (*n* = 20, 51.3%) were classified as middle wealth, compared to participants in the AUD treatment cohort (*n* = 43, 28.9%; *p* = 0.036). Participants in the treatment group also reported greater AUDIT scores (U = -7.46; *p* < 0.001) and PHQ-9 scores (U = -4.87; *p* < 0.001) compared to participants in the control group (see Table 1).

The attrition rate in the depression cohort was 29.8% (*n* = 159) at midline and 31.1% (*n* = 166) at endline. In the AUD cohort, the attrition rate was 28.5% (*n* = 163) and 30.9% (*n* = 177) at midline and endline, respectively. Participants who were followed up at midline and those lost to attrition did not differ on baseline demographic or clinical characteristics in the depression cohort. They did, however, differ on age and AUDIT scores. At endline, those lost to follow-up in the AUD cohort reported lower baseline AUDIT scores (median = 25, IQR = 20–19) compared to those followed-up (median = 21, IQR = 14–28, *p* = 0.010). Finally, a greater proportion of those lost to follow-up at endline in the depression cohort reported a higher education (*n* = 26, 60.5%) and a smaller proportion reported being Hindu (*n* = 31, 72.1%) compared to those assessed at endline (education: *n* = 70, 42.2%, *p* = 0.042; religion: *n* = 145, 87.4, *p* = 0.020). There were no other differences, and so missing data were considered missing at random.

The results of the negative binomial regression models to assess the effect of receiving treatment in the depression and AUD cohorts, adjusted for baseline differences, are presented in Table 2. The adjusted mean changes in PHQ-9 scores between baseline and midline or endline were not significantly different between the treatment (TG and TG + P) and control groups (TAU) in the depression cohort. However, adjusted mean reductions in WHODAS scores among participants in the treatment groups were marginally greater at midline (adjusted β = − 6.62; 95%CI − 13.86 to 0.63; *p* = 0.073) and endline (aβ = − 7.20; 95%CI − 14.75 to 0.35; *p* = 0.062) compared to participants in the control group. In the AUD cohort, adjusted mean change in AUDIT Scores were significantly greater among the participants in the treatment groups compared to the control group, at both midline (aβ = − 15.13; 95%CI − 18.63 to − 11.63; *p* < 0.001) and endline (aβ = − 9.12; 95%CI − 13.41 to − 4.83; *p* < 0.001). Adjusted mean change in WHODAS scores were also greater for the treatment groups at endline (aβ = − 9.26; 95%CI − 16.41 to − 2.12; *p* = 0.011). The difference between both groups was only marginal at midline (aβ = − 6.26; 95%CI − 12.95 to 0.43; *p* = 0.067).

**Table 2:** Impact of PRIME mental health care plan on individual level outcomes

	Control group			Treatment group			Adjusted β (95%CI)	*P*	Effect size (95%CI)
	N	Mean (SD)	Adjusted mean change (95%CI) from BL	N	Mean (SD)	Adjusted mean change (95%CI) from BL			
**Depression Cohort**									
PHQ-9									
Baseline	72	12.3 (2.15)	–	137	12.1 (4.13)	–	–	–	
Midline	49	6.3 (5.06)	−6.22 (−7.64 to −4.79)	110	4.7 (4.32)	−7.09 (−8.17 to − 6.02)	−0.88 (−2.66 to 0.91)	0.336	− 0.17 (− 0.50, 0.17)
Endline	55	6.6 (4.94)	−5.53 (− 6.91 to − 4.14)	111	4.8 (4.60)	−6.93 (− 8.07 to − 5.78)	−1.40 (−3.20 to 0.40)	0.127	−0.25 (− 0.57, 0.07)
WHODAS									
Baseline	72	30.0 (15.55)	–	137	35.7 (18.55)	–	–	–	
Midline	49	21.7 (18.50)	−8.08 (− 13.62 to − 2.54)	110	18.9 (18.83)	−14.70 (− 19.37 to − 10.03)	−6.62 (− 13.86 to 0.63)	0.073	−0.31 (− 0.64, 0.03)
Endline	55	22.8 (18.89)	−6.71 (− 12.28 to − 1.13)	111	19.8 (20.92)	− 13.90 (− 19.00 to − 8.81)	− 7.20 (− 14.75 to 0.35)	0.062	− 0.31 (− 0.63, 0.02)
**AUD Cohort**									
AUDIT									
Baseline	57	16.4 (6.53)	–	175	25.4 (6.55)	–	–	–	
Midline	28	16.0 (5.92)	1.66 (−0.83 to 4.15)	135	10.4 (9.87)	−13.48 (− 15.94 to − 11.01)	−15.13 (− 18.63 to − 11.63)	< 0.001	−1.76 (− 2.17, − 1.35)
Endline	35	16.9 (9.04)	1.09 (− 2.36 to 4.54)	142	15.7 (10.74)	− 8.03 (− 10.59 to − 5.48)	−9.12 (− 13.41 to − 4.83)	< 0.001	− 0.79 (− 1.16, − 0.42)
WHODAS									
Baseline	57	17.5 (15.11)	–	175	22.1 (17.90)	–	–	–	
Midline	28	13.4 (12.58)	− 2.57 (− 8.00 to 2.86)	135	12.3 (15.77)	− 8.83 (− 12.75 to − 4.92)	− 6.26 (− 12.95 to 0.43)	0.067	−0.38 (− 0.79, 0.03)
Endline	35	15.5 (13.03)	1.23 (− 4.82 to 7.28)	142	13.6 (14.33)	−8.03 (− 11.83 to − 4.23)	− 9.26 (− 16.41 to − 2.12)	0.011	− 0.48 (− 0.85, − 0.11)

*AUDIT* Alcohol use disorder identification test, *BL* Baseline, *PHQ-9* Patient Health Questionnaire – 9 item, *SD* Standard deviation, *WHODAS* WHO Disability Assessment Schedule

Similar results were found for the AUD cohort when only participants who received CAP were considered in the treatment groups (TG and TG + P) (see Table 3). However, in the depression cohort, post-hoc analyses showed that when only participants who received HAP were considered in the treatment groups, these participants showed a significantly greater mean change in PHQ-9 score at midline (adjusted β = −-2.64; 95%CI − 4.55 to − 0.74; *p* = 0.007) and endline (aβ = − 3.66; 95%CI − 5.55 to − 1.77; *p* < 0.001) compared to participants in the control group. This was also the case for WHODAS scores at midline (aβ = − 12.20; 95%CI − 19.79 to − 4.62; *p* = 0.002) and endline (aβ = − 14.26; 95%CI − 22.65 to − 5.86; *p* = 0.001).

**Table 3:** Post-hoc analysis of impact of PRIME mental health care plan on individual level outcomes, excluding treatment participant who only received basic MHCP intervention

	Control group			Counselling (HAP/CAP) group			Adjusted β (95%CI)	*P*	Effect size
	N	Mean (SD) or N (%)	Adjusted mean change (95%CI) from BL	N	Mean (SD) or N (%)	Adjusted mean change (95%CI) from BL			
**Depression Cohort**									
PHQ-9									
Baseline	72	12.3 (2.15)	–	60	13.0 (3.37)	–	–		
Midline	49	6.3 (5.06)	−6.33 (−7.77 to −4.89)	46	3.9 (3.56)	−8.97 (−10.21 to − 7.72)	−2.64 (− 4.55 to −0.74)	0.007	− 0.56 (− 0.96, − 0.16)
Endline	55	6.6 (4.94)	−5.50 (− 6.90 to − 4.11)	46	3.4 (2.95)	−9.16 (− 10.44 to − 7.89)	−3.66 (− 5.55 to − 1.77)	< 0.001	− 0.76 (− 1.15, − 0.37)
WHODAS									
Baseline	72	30.0 (15.55)	–	60	36.5 (15.50)	–	–		
Midline	49	21.7 (18.50)	− 6.89 (− 12.43 to − 1.35)	46	14.1 (15.81)	−19.09 (− 24.27 to − 13.91)	− 12.20 (− 19.79 to − 4.62)	0.002	−0.65 (− 1.05, − 0.25)
Endline	55	22.8 (18.89)	−5.78 (− 11.34 to − 0.22)	46	13.1 (15.35)	−20.04 (− 26.33 to − 13.75)	−14.26 (− 22.65 to − 5.86)	0.001	−0.67 (− 1.06, − 0.27)
**AUD Cohort**									
AUDIT									
Baseline	57	16.4 (6.53)	–	80	26.0 (6.46)	–	–		
Midline	28	16.0 (5.92)	3.78 (0.57 to 6.98)	63	10.1 (9.55)	−13.96 (− 17.88 to − 10.04)	− 17.74 (− 22.80 to − 12.67)	< 0.001	− 1.56 (− 2.00, − 1.11)
Endline	35	16.9 (9.04)	2.75 (−1.72 to 7.22)	66	14.9 (11.08)	−7.69 (− 12.30 to − 3.08)	− 10.45 (− 16.87 to − 4.02)	0.001	− 0.67 (− 1.08, − 0.26)
WHODAS									
Baseline	57	17.5 (15.11)	–	80	22.8 (18.64)	–	–		
Midline	28	13.4 (12.58)	−2.43 (− 8.09 to 3.24)	63	11.7 (14.37)	−9.87 (− 15.15 to − 4.58)	−7.44 (− 15.19 to 0.31)	0.060	−0.43 (− 0.87, 0.02)
Endline	35	15.5 (13.03)	4.68 (− 2.97 to 12.34)	66	12.6 (14.14)	− 8.39 (−14.03 to − 2.75)	−13.08 (− 22.58 to − 3.57)	0.007	−0.56 (− 0.97, − 0.15)

The inverse probability weights were recalculated based on baseline differences between the control and refined treatment groups in the depression and AUD cohorts, hence adjusted mean change values differ from Table 2

*AUDIT* Alcohol use disorder identification test, *BL* Baseline, *PHQ-9* Patient Health Questionnaire – 9 item, *RR* Risk ratio, *SD* Standard deviation, *WHODAS* WHO Disability Assessment Schedule

**Fig. 3:**
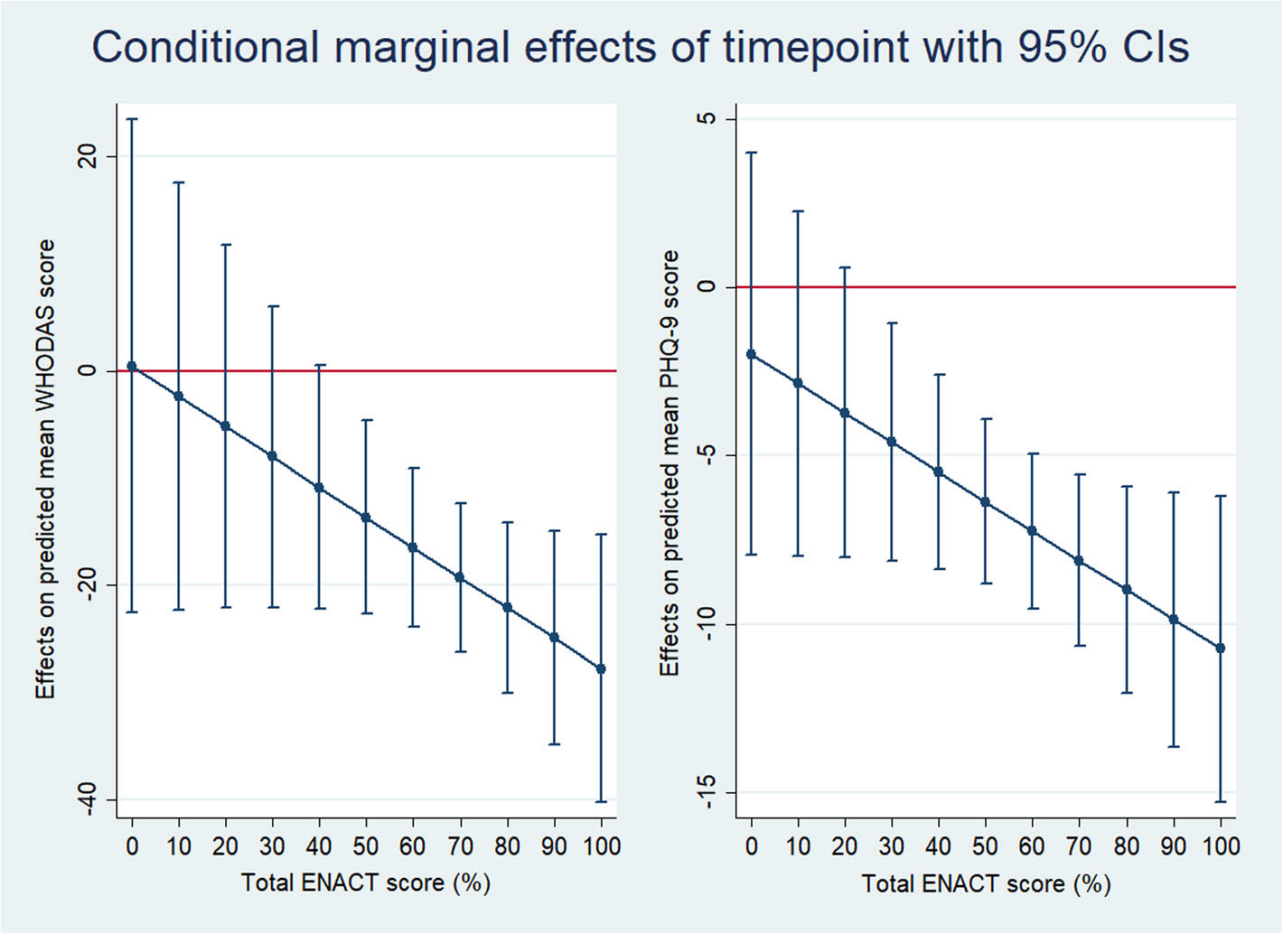
Difference in WHODAS and PHQ-9 scores between baseline and endline across patient-rated ENACT scores

Results from models assessing perceived quality of care (PACIC) as a predictor of outcomes indicate that there was no association of PACIC score over time in predicting WHODAS scores (β = − 0.14; 95%CI − 0.45 to 0.18, *p* = 0.389) or PHQ-9 scores (β = − 0.03; 95%CI − 0.13 to 0.07, *p* = 0.607) in the depression treatment cohort. Analyses of the association of patients' perceptions of health worker competency (patient-ENACT) on functioning and symptom severity among participants in the depression cohort indicate that there was a marginal effect for ENACT scores in predicting the WHODAS (β = − 0.28; 95%CI − 0.61 to 0.04; *p* = 0.088); at endline, WHODAS scores decrease significantly as ENACT scores increase (β = − 0.36; 95%CI − 0.55 to − 0.18; *p* < 0.001). The interaction between ENACT and time in predicting WHODAS scores is significant when ENACT scores are at or above 41 (see Fig. 3). In other words, the difference in WHODAS scores between baseline and endline only becomes significant when ENACT scores are at least 41.

A similar pattern can be seen for PHQ-9 scores (β = − 0.09; 95%CI − 0.18 to 0.01; *p* = 0.069). The overall association is not significant at endline (β = − 0.03; 95%CI − 0.08 to 0.01, *p* = 0.102). Further investigation of indicate that the interaction between ENACT and time in predicting PHQ-9 scores is significant when ENACT scores are at or above 25 (see Fig. 3).

Finally, analyses indicate that there is no association of ENACT in predicting WHODAS (β = − 0.05; 95%CI − 0.23 to 0.14, *p* = 0.637) or AUDIT scores (β = − 0.03; 95%CI − 0.27 to 0.20, *p* = 0.782) over time in the AUD treatment cohort. PACIC scores are not associated with either WHODAS (β = − 0.03; 95%CI − 0.16 to 0.23, *p* = 0.729) or AUDIT scores (β = − 0.04; 95%CI − 0.19 to 0.27, *p* = 0.753) over time.

## Discussion

In a previous study we have demonstrated that primary healthcare workers implementing the mhGAP-based treatment for people diagnosed with depression and AUD resulted in significant improvements in clinical and functioning outcomes, 12 month after initiation of treatment [29]. While our earlier finding confirms that briefly trained primary healthcare workers implementing evidence-based mental health services result in the expected outcomes, it does not provide insight into the added value of these services compared to healthcare as usual or what factors might explain changes in patient outcomes.

The current study used intervention cohorts of people diagnosed with depression and AUD by primary healthcare workers. The results demonstrate that for people with depression there is no significant difference between those receiving healthcare as usual compared to those receiving additional mental health services – both see similar improvements over time. Findings from our previous pragmatic randomized controlled trial demonstrated that receiving a non-specialist counsellor delivered psychological treatment (i.e., HAP) in addition to the primary healthcare mental health services resulted in superior outcomes for people with depression [14]. In the current study, we therefore compared the subgroup who received mental health services that included counselling with healthcare as usual. Results demonstrated significantly better clinical and functional outcomes for the group also receiving a psychological treatment.

Based on the combination of these results, we conclude that the treatment benefits for depression appear to be explained by patients receiving the psychological treatment. It appears that providing regular health care for somatic complaints (the presumed reason for help seeking for most study participants), has an impact on the reduction of depression symptoms. This is not surprising given the well-established association between improved physical health and mental health [30]. In order to make treatment for depression in primary care meaningful, a psychological treatment should be offered. While there are only a limited number of trials on depression treatment in primary care in LMIC, there is evidence that psychological treatments for depression are effective in primary care [31]. These findings are also consistent with a study in India that demonstrated that a collaborative care intervention by non-specialist counsellors can lead to an improvement in recovery from common mental disorders among patients attending public primary care facilities [32].

For people diagnosed by health workers with AUD, the results demonstrate that mhGAP-based mental health services, with or without adding a psychological treatment (i.e. CAP), resulted in significantly better clinical and functioning outcomes when compared to healthcare as usual. Given that the above-mentioned pragmatic trial demonstrated that there was no significant difference in outcomes between the group that received health worker provided mental health services and the group that received additional community counsellor-delivered psychological treatment, we conclude that the treatment benefits for AUD appear to be explained by the primary healthcare workers implementing the mhGAP guidelines. This is congruent with available evidence from LMIC demonstrating the effectiveness of brief intervention and pharmacotherapy, both of which are included in the mhGAP training the health workers received, in reducing alcohol consumption to low-risk levels among hazardous and harmful drinkers [33].

The results on predictors of treatment effect for people with depression and AUD indicate that patient-perceived quality of chronic illness care is not associated to better outcome in either group. The absence of an association between perceived quality of patient-centred care for chronic illness and outcomes can have two explanations. First, the study was underpowered to demonstrate predictive relations. Second, the PACIC asked for elements of quality of care that were not explicitly part of the training of health workers. On the other hand, patient’s perception of health workers’ skills in common factors (empathy, communication skills, etc.) appears to predict better clinical and functioning outcomes. Patients reporting greater aptitude in common factors for their health workers were more likely to display greater depression treatment effect. Patients' perceptions of health worker aptitude in common factors is a significant predictor once a minimum, or base, level of perceived common factors is reached. The specific ENACT scores that indicated the tipping point for being a predictor for outcomes should not be seen as actual cut-off scores, rather as indication of a trend that higher perceived competencies predict better outcomes. For people with AUD, this association was not found. Service providers’ aptitude in common factors is important because it is an indicator of quality of care and is an important factor in the standardization of the implementation of evidence-based care [34]. In this case it can be hypothesized that high patient-ratings of health workers competencies is a proxy for their trust in the provider, or in the quality of received treatment – speaking to the importance of common therapeutic factors [35]. The ENACT has been developed to assess service providers’ competence after receiving training, as well as their ability to deliver mental health care [27, 36]. The use of the ENACT aims to contribute to improving quality of care, as training and supervision can remediate where competences falls short. This study uniquely shows the promise of patient-reported competencies as a service-user led evaluation of satisfaction with, and perceived quality of mental of, mental health services. Moreover, it may present with a less time-intensive alternative, or addition, to supervisor-observed assessments. This is compatible with the growing recognition of increasing service user involvement in mental health care planning and evaluation [37].

### Implications

Based on the results of this study, we can formulate a number of recommendations for future efforts to integrate mental health care in primary healthcare. First, training health workers in the depression module of mhGAP might not be a worthwhile strategy. Rather, people with depression should receive an evidence-based psychological treatment, such as behaviour activation based HAP, to achieve better treatment effects than healthcare as usual. Second, for people with AUD the picture is the reverse. Based on our results, it does not appear to be a good strategy to invest in offering a psychological treatment. Rather, health workers should be trained to implement the mhGAP module on AUD as part of their routine work. Third, instead of monitoring patient overall perceived quality of care, which is not associated with outcomes, specifically monitoring the patients’ perception of the health workers’ clinical competencies might be an important indicator to track for depression care. Especially if a minimum threshold level of perceived competency is established, then the monitoring of this indicator can become an important tool for supervision and quality improvement of mental health care.

### Limitations

There are a few limitations that should be taken into account when interpreting the findings from this study. First, the treatment groups consisted of those screened positive and diagnosed by a health worker, whereas the control group consisted of screened positives only. This difference might have resulted in the treatment group including participants reporting higher levels of symptom severity, potentially impacting estimates of treatment effectiveness. Furthermore, we observed distinctly more so-called high-caste and fewer Dalit participants in the treatment groups (TG and TG + P) compared to the control group (TAU). This trend is congruent with previous studies demonstrating more negative outcomes among Dalit participants when compared to high caste participants [38–40]. In the present study this can be explained by a tendency for Dalit patients to feel uncomfortable to share mental health problems with mostly high-caste health workers, combined with stigmatizing attitudes by health workers towards Dalits. The results on treatment benefits should be interpreted with caution, as the allocation between treatment groups and control group was not done at random. Second, the target sample size was not achieved due to lower than expected client flow during the recruitment period. The study was not powered to evaluate mediation or moderation effects. The presented results on predictors of outcomes should therefore be seen as exploratory in nature. Third, the non-diagnosed control group may have still received mental health services outside the primary health centers. While this is unlikely, given the scarcity of mental health care, it is possible.

## Conclusion

Efforts to integrate mental health into primary health care should emphasize the availability of psychological treatment for people with depression. For people presenting with AUD, the training of health workers in implementing the mhGAP module should be emphasized. Perceptions of competencies of the service provider appears to be a predictor of treatment outcomes for depression. As this has the potential to be become a useful monitoring indicator, this should be tested as an a priori hypothesis in future research.

## Data Availability

The data supporting the findings will be made available through the PRIME program’s website http://www.prime.uct.ac.za/.

## Abbreviations

AUD: Alcohol use disorder
TG: Treatment Group
TG + P: Treatment Group plus Psychological treatment
TAU: Treatment as usual
WHO: World Health Organization
LMIC: Low- and Middle Income Countries
mhGAP: Mental health Gap Action Programme
PRIME: Programme for Improving Mental Health Care
HAP: Healthy Activity Programme
CAP: Counselling for Alcohol Problems
MHCP: Mental health care plan
PHQ: Patient Health Questionnaire (− 9) [1]
AUDIT: Alcohol Use Disorder Identification Test
WHODAS: WHO Disability Assessment Schedule
IRT: Item Response Theory
PACIC: Patient Assessment on Chronic Illness Care
ENACT: ENhancing Assessment of Common Therapeutic factors
IQR: Inter-Quartile Range
